# Editorial: Suicide and related behaviour, volume II

**DOI:** 10.3389/fpsyt.2024.1462051

**Published:** 2024-08-09

**Authors:** Gonzalo Martínez-Alés, Jorge Lopez-Castroman, Maria Luisa Barrigón, Enrique Baca-Garcia

**Affiliations:** ^1^ Icahn School of Medicine at Mount Sinai, New York, NY, United States; ^2^ Harvard TH Chan School of Public Health, Boston, MA, United States; ^3^ Centro de Investigación Biomédica en Red de Salud Mental, Madrid, Spain; ^4^ Hospital La Paz Research Institute (IdiPAZ), Madrid, Spain; ^5^ Department of Psychiatry, Radiology, Public Health, Nursing and Medicine, University of Santiago de Compostela, Santiago de Compostela, Spain; ^6^ Institut National de la Santé et de la Recherche Médicale (INSERM), University of Montpellier, Neuropsychiatry: Epidemiological and Clinical Research, Montpellier, France; ^7^ Department of Adult Psychiatry, Nimes University Hospital, Nimes, France; ^8^ Department of Adult Psychiatry, Hospital General Universitario Gregorio Marañon, Madrid, Spain; ^9^ Department of Psychiatry, Hospital Universitario Fundacion Jimenez Diaz, Madrid, Spain; ^10^ Department of Psychiatry, Universidad Autonoma de Madrid, Madrid, Spain

**Keywords:** suicide, suicidal ideation, parasuicide, NSSI deliberate self-harm, suicidal behavior

Suicide continues to be the leading cause of violent death. While suicide mortality decreased globally by 30% between 1990 and 2020, largely driven by dramatic declines in suicide rates in China and India, suicide trends vary markedly across place and sociodemographic groups. In fact, suicide mortality increased during by over 30% in a variety of locations – e.g., Jamaica, Mexico, or the United States after 1990; and recent studies indicate ongoing increasing suicide trends in youth, females, and socioeconomically minoritized groups across the globe. Ensuring access to research conducted in different social contexts and with a focus on high-risk sociodemographic groups is paramount to advance understanding of drivers of suicide and inform prevention strategies.

Major scientific contributions to suicide prevention efforts over the last decades have yielded a rich evidence base on effective interventions to guide healthcare planning and delivery. There is now an accumulating body of knowledge on public health and clinical interventions to increase safety for individuals experiencing suicidal crises (e.g., through reduction in access to lethal means or easing access to healthcare). Translation of this evidence into real-world action, though, remains challenging: research on specific implementation aspects of evidence-based interventions, including on attitudinal and economic barriers and facilitators to program deployment, continues to be a major public health and clinical need.

In this special topic on Suicide and Related Behavior, we sought to include research advancing knowledge on the drivers of suicide risk among risk populations through a global lens – emphasizing the need to increase the presence of researchers from locations traditionally underrepresented in the scientific literature. We also aimed at selecting research on the real-world implementation of evidence-based suicide prevention strategies, to bridge the gap between research and public health decision-making. Here we provide a brief overview of the contents and highlights of the special topic.

Two studies examined social and clinical factors associated with suicidal thoughts and behaviors among youth. Veloso-Besio et al. found that, of 728 Chilean individuals aged an average 15.6 years from the Arica region, almost 20% had attempted suicide and more than 60% had experienced suicidal ideation – with even higher prevalence among females. In a set of regression analyses, they found relevant associations between suicidal thoughts and behaviors and bullying experiences. Along these lines, a study by Wang et al. used a sample of more than 1300 Chinese college students to examine whether two domains pertaining to cognitive style (experiential avoidance and rumination) and depression mediated the association between reported childhood emotional abuse and suicidal ideation and behaviors, finding that all three proposed mediators had a role in the link under study. While these two studies were cross-sectional and relied on major assumptions (for instance, use of a convenience sample likely explains some findings such as the remarkable high rates of suicidal ideation in the latter study), they help identify potential targets for intervention to reduce suicide risk in adolescents, a demographic group with recent, concerning increases in suicidal behaviors and suicide mortality globally.

An additional set of two studies focused on other demographic and clinical groups at increased suicide risk: individuals living with bipolar disorder and older adults. The systematic review by Garcia-Jimenez et al. sought to clarify if the association between cigarette smoking and suicide that has previously been reported in unselected individuals can also be found among bipolar disorder patients, who have higher risks of both smoking and suicide than the general population. High heterogeneity across studies in both sample selection and variable definition likely explains their finding that an association between smoking and suicidal behaviors among bipolar disorder patients was only present in 9 out of 15 included studies – not lending itself to a straightforward interpretation and highlighting the need for further research using systematic approaches to measurement and high-quality clinical cohorts. A paper including over 600 suicide attempters from Wonju, in South Korea, attempted to advance understanding of factors associated with increased attempt lethality among older adults: they found that suicide attempts following death of a family members may entail the lowest lethality among different potential motivations for suicide, and that lethality of attempt increased with age and, unsurprisingly, with reported intent to die.

Three articles explicitly examined aspects related to the implementation of suicide prevention strategies in the real world. Substantial evidence indicates that, for most suicide attempters, increasing contact with providers following discharge after an attempt can reduce risk of subsequent suicidal behaviors, especially during the initial weeks as re-attempt risk is highest shortly after a suicide attempt. Examining health records on 554 individuals who received extra-hospital care after a suicide attempt in Malaga, Spain, Ramos-Martin et al. found that patients who were initially referred to primary care had a higher likelihood of disengagement from care than counterparts referred directly to mental health providers. Two studies from the United States focused on two interventions with major scientific traction at the public health and clinical levels: Zero Suicide strategies and the Safety Planning Intervention. Jasperson et al., in a system-wide survey administered within the University of Utah Health System, found that a remarkable low proportion of respondents (less than 10%) had undergone training on Counseling on Access to Lethal Means, an important component of Zero Suicide initiatives, despite the fact than more than half of the respondents reported directly interactions with individuals who may be at suicide risk. They also found associations between completion of training on lethal means counseling and (i) knowledge on suicide warning signs and (ii) confidence on ability to respond to suicide risk situations – suggesting that training on lethal means counseling may be related to higher levels of empowerment and agency when facing suicide risk in the workplace. Raciborski et al. took advantage of an ongoing randomized trial in a United States Veterans population and of the initial pandemic outbreak to examine, using a natural experiment design, the comparative cost of in-person and telehealth Safety Planning augmented with Project Life Force – a manualized group intervention to maximize use of the safety plan during suicidal crisis. While the authors did not report effectiveness estimates, they found that the telehealth-based group intervention had similar adherence and lower costs, resulting in overall savings, than the in-person comparison. Overall, these three studies indicate that low-cost, easy to implement interventions (i.e., direct referral to mental health rather than primary care providers following a suicide attempt, training on Counseling on Access to Lethal Means, and a telehealth-based group to augment use of a safety plan) may improve health system performance in terms of prevention of suicidal behaviors while also contributing to health system economic sustainability.

Last, a stakeholder analysis by Balt et al. including the input of persons with personal experience and bereaved by suicide alongside healthcare, policy, and research leaders highlighted current limitations in implementation of standardized procedures for psychological autopsies, defining a path forward to advance much needed understanding of the causes and impact of deaths by suicide.

Most suicides are preventable. We thank the authors featured in our special topic for contributing to the advancement of knowledge on suicidal behaviors, their underlying mechanisms, and the real-world implementation of evidence-based interventions for their prevention. This research should generate interest and vigorous debate among scientists and stakeholders, and continue to stimulate further efforts to guide public health and clinical decision-making for suicide prevention.

